# Clinical, EEG and neuroimaging features of ictal epileptic headache

**DOI:** 10.1186/1129-2377-14-S1-P156

**Published:** 2013-02-21

**Authors:** NZR Radojkovic Gligic, KKK Kacar, SMA Atic, BVV Vekic

**Affiliations:** 1 Hospital for cerebrovascular desease St. Sava, Serbia and Montenegro

## Introduction

Migraine end epilepsy have common pathophysiologic mechanisms and share essential and defining attributes which distinguish them from other common neurological disorders. They are both characterized by paroxysmal symptoms and are, therefore, episodic disorders. Occipital lobe to be the brain structures most responsible for development of migraine and occipital lobe epilepsies Both are characterized by visual symptoms followed by headache and other autonomic symptoms. Recognition of headache as an epileptic manifestation per se still represents a challenge.

## Methods

This study included consecutive 89 patients with IEH= ictal epileptic headache,at our hospital and our diagnostical department – MRI and EEG.

## Results

EEG recordings high voltage rhythmic 11-12 Hz activity with intermingled spikes over the right TO regions. Other –high voltage theta activity intermingled with sharp waves over occipital region. And third bilateral SWC.Brain MRI showed secondary brain lesion in the right TPO region with restricted diffusion in the right occipital region.

## Conclusion

IEH be used to classify the events in wich headache represent the only ictal epileptic feature These rare cases should be classified as autonomic epilepsy.
